# The Spectrum of HIV‐Associated Kidney Pathology at a Tertiary Centre in South Africa

**DOI:** 10.1155/ijne/3219794

**Published:** 2026-07-30

**Authors:** Anna-Liisa Tonata Kaapangelwa, Thabiet Jardine, Liezel Coetzee, Mogamat-Yazied Chothia

**Affiliations:** ^1^ Division of Nephrology, Department of Medicine, Faculty of Medicine and Health Sciences, Stellenbosch University and Tygerberg Hospital, Cape Town, South Africa, sun.ac.za; ^2^ Division of Anatomical Pathology, Department of Pathology, Faculty of Medicine and Health Sciences, Tygerberg Hospital, Stellenbosch University and National Health Laboratory Service, Cape Town, South Africa, upm.edu.my

**Keywords:** biopsy, HIV, kidney, kidney diseases, South Africa

## Abstract

**Aim:**

South Africa has one of the highest prevalences of human immunodeficiency virus (HIV) infection worldwide. Kidney disease is a common and important complication of HIV. This study describes biopsy‐proven kidney pathology in people living with HIV (PLHIV) over 29 years at a South African centre, comparing patterns before and after antiretroviral therapy (ART) rollout.

**Methods:**

This retrospective descriptive study included PLHIV who underwent native kidney biopsy at Tygerberg Hospital during the period of 1 January 1992 to 31 December 2020. Patients were categorised by biopsy year into a pre‐ART rollout period (before 2004) and a post‐ART rollout period (2004 or later).

**Results:**

The cohort included 554 PLHIV, mostly biopsied post‐ART rollout (90.1%). Mean age was 36.5 years, with a balanced sex distribution. During the post‐ART period, 54.8% were on ART at biopsy. HIVAN was diagnosed in 45.7%, of which one‐third had an additional histopathological pattern of injury. In the pre‐ART rollout period, HIVAN was the most prevalent diagnosis (60.0%), whereas a lower prevalence was observed post‐ART rollout (44.1%). During the post‐ART rollout period, more than half of patients (55.9%) exhibited only non‐HIVAN kidney pathology, with immune‐complex‐mediated glomerulonephritis (52.7%) and tubulointerstitial disease (39.7%) the most common.

**Conclusion:**

This study describes the spectrum of kidney pathology in PLHIV at a South African tertiary centre over nearly three decades, highlighting a significant HIVAN burden overall. We observed a lower proportion of patients with HIVAN following ART rollout, coinciding with an increase in non‐HIVAN pathologies like immune complex glomerulonephritis and tubulointerstitial disease.

**Summary**
 This retrospective study describes the spectrum of biopsy‐proven HIV‐associated kidney pathology at a tertiary centre in South Africa over nearly three decades. Although HIV‐associated nephropathy (HIVAN) remained highly prevalent overall, the period after the rollout of ART was characterised by a decline in the prevalence of HIVAN and a corresponding increase in the prevalence of non‐HIVAN kidney pathology.

## 1. Introduction

**FIGURE 1:**
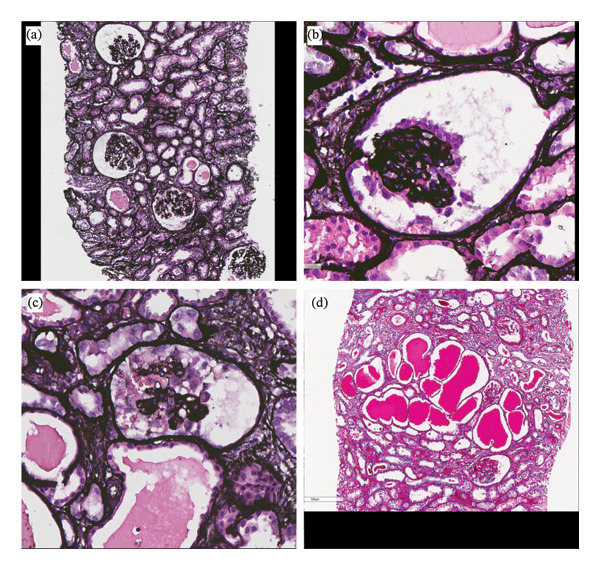
Histological features of HIVAN. (a) Low‐power section of the Jones silver stain showing collapsing glomerulopathy characteristic of HIVAN. (b) Higher‐power view of a collapsed glomerular tuft with an associated increase in Bowman’s space (Jones silver stain). (c) Segmental collapse of the glomerular tuft and hyperplasia of overlying visceral epithelial cells containing cytoplasmic protein droplets (Jones silver stain). (d) Periodic acid‐Schiff‐stained sections demonstrating cystic changes of the tubules containing proteinaceous material.

South Africa has one of the highest prevalences of HIV in the world; more than 5.7 million South African adults are estimated to be living with HIV, with a reported prevalence of 19% among economically active adults [1]. Kidney disease is an important complication of HIV infection, which increases the risk of both acute kidney injury (AKI) and chronic kidney disease (CKD) [2]. Furthermore, kidney disease is associated with increased morbidity and mortality among those living with HIV [3, 4]. The pathogenesis of kidney disease in HIV‐infected individuals is complex and multifactorial, with postulated mechanisms including direct cytotoxic effects of the virus on renal epithelial cells and immune‐mediated injury (including complex‐mediated deposition). Certain genetic polymorphisms, most notably high‐risk variants of the APOL1 gene, may predispose individuals of African ancestry to kidney disease in the setting of HIV infection. In addition, antiretroviral therapy (ART)‐associated nephrotoxicity, particularly related to tenofovir disoproxil fumarate (TDF), Hepatitis B and C virus co‐infection and comorbidities such as diabetes mellitus and hypertension—which have become increasingly common among patients living with HIV due to improved survival and ART‐related metabolic side effects—may further contribute to the development of kidney disease in this population [4, 5]. Indeed, a wide spectrum of kidney pathology may occur in people living with HIV, with HIVAN being the most well‐characterised [6]. This condition results from virus‐induced damage and dedifferentiation of podocytes, leading to their shedding into Bowman’s space and collapse of the glomerular tuft [1, 5]. Clinically, untreated HIVAN presents with impaired kidney function, often with a rapid progression to kidney failure, nephrotic‐range proteinuria without hypertension or oedema and large kidneys on ultrasonography [1, 5]. Histologically, it is characterised by a type of collapsing focal segmental glomerulosclerosis (FSGS), together with tubulocystic dilatation and interstitial damage (Figure 1) [4]. In some studies, female sex, low CD4 T‐helper cell numbers and high viral loads are associated with an increased risk of developing HIVAN [4, 7]. In contrast to HIVAN, FSGS (not otherwise specified, NOS) in the setting of HIV is characterised by discrete segmental scars and segmental adhesions to Bowman’s capsule, without features of collapse, glomerular epithelial cell hyperplasia or typical interstitial changes [8].

Historically, HIVAN was the most observed kidney disease in people living with HIV in South Africa [5, 6]. In a study conducted at a South African hospital prior to the rollout of ART, the prevalence of HIVAN was estimated at 83% among HIV‐infected individuals being evaluated for proteinuria [5]. Other pathologies observed in the setting of HIV include various immune complex glomerulonephritides (formerly known as HIV‐associated immune complex disease or ‘HIVICK’), nonspecific mesangial proliferative glomerulonephritis, immunoglobulin A nephropathy (IgAN), postinfectious glomerulonephritis (PIGN), membranous nephropathy, interstitial nephritis, FSGS and, less commonly and thrombotic microangiopathy (TMA) [5, 6, 9, 10].

Following the rollout of ART, changes in the patterns of kidney pathology among people living with HIV in South Africa have been observed [6, 9, 11]. Several South African centres have reported a declining prevalence of HIVAN following ART rollout, with a concomitant increase in patients with non‐HIVAN pathology [11–14].

In this study, we aim to describe the spectrum of kidney pathology in people living with HIV who underwent native kidney biopsy at Tygerberg Hospital, a tertiary centre in Cape Town, South Africa, over a 29 year period. We further examine changes in disease patterns in relation to the rollout of ART in 2004 and identify the most common non‐HIVAN pathologies in the cohort.

## 2. Methods

This retrospective, descriptive study included people living with HIV who underwent native kidney biopsy at Tygerberg Hospital, Cape Town, South Africa, during the period of 1 January 1992 to 31 December 2020. Our main data source was the institutional kidney biopsy registry. Where folder numbers were available, we also used the National Health Laboratory Service (NHLS). Only adequate biopsies were included.

The rollout of ART in South Africa officially commenced in April 2004 [15]. In this study, patients were categorised by biopsy year into a pre‐ART rollout period (before 2004) and a post‐ART rollout period (2004 or later). Pathologic patterns and covariates were compared across the two groups.

We described the histopathological patterns of kidney injury, with patients categorised into one of three broad pathological groups: isolated HIVAN only (HIVAN only), HIVAN with at least one other histopathological pattern of kidney injury (HIVAN with additional pathology) or non‐HIVAN pathology only. For those with HIVAN with additional pathology or non‐HIVAN pathology only, we described the additional histopathological patterns.

Histopathological patterns described broadly align with the Kidney Disease: Improving Global Outcomes (KDIGO) classification of kidney disease in the setting of HIV. As such, in this study, immune‐complex‐mediated glomerulonephritis includes PIGN, mesangiocapillary (MCGN, also known as membranoproliferative glomerulonephritis), lupus nephritis, lupus‐like glomerulonephritis in the setting of HIV, membranous nephropathy, mesangial proliferative glomerulonephritis and IgA nephropathy, as well as any other immune complex kidney disease in the setting of HIV. Tubulointerstitial disease (TID or tubulointerstitial dominant pattern of disease) comprises acute tubular necrosis (ATN), tubulointerstitial nephritis (TIN) from any cause and pyelonephritis [16].

We collected the following clinic‐demographic and laboratory variables: age, sex, blood pressure, serum creatinine, proteinuria (g/day), CD4 count, ART use at the time of biopsy and Hepatitis B status. Proteinuria was obtained from the kidney biopsy registry, where it is recorded as a clinician‐determined numeric estimate (g/day). The method of proteinuria quantification (spot urine protein‐to‐creatinine ratio or 24 h urine collection) was not recorded in the registry. Viral load was not included among the collected variables.

### 2.1. Statistical Analysis

Data were analysed using Stata Version 19.5 (StataCorp LLC, Texas, USA). Data with a normal distribution are shown as means with standard deviations, whereas non‐normally distributed data are presented as medians with interquartile ranges. Continuous variables following a normal distribution were compared using Student’s *t*‐test, while non‐normal variables were compared using the Mann–Whitney *U* test. We used chi‐squared or Fisher’s exact test to compare categorical variables. A *p* value less than 0.05 was regarded as statistically significant, and 95% confidence intervals were employed.

### 2.2. Handling of Missing Data, Multiple Imputation and Sensitivity Analysis

Missing data were assumed to be missing at random (MAR) following analysis of patterns of missingness and associations of missing variables with observed variables. We performed multiple imputation with chained equations (MICE, *m* = 40) to handle variables with missingness of up to 50%. Variables with a high level of missingness (> 50%) in one period were considered structurally missing and were excluded from imputation for that specific era. After multiple imputation, an exploratory multinomial regression was carried out to assess whether there was an association between ART use and kidney pathology, following adjustment for the case mix. Adjusted odds ratios, accompanied by 95% confidence intervals, are presented to quantify the effect of the ART use on the histopathological pattern. A mixed‐pattern sensitivity analysis using the delta method was performed because we could not definitively rule out missing data that were not MAR (NMAR). Furthermore, complete case analysis was done, including only patients with complete observations across all variables.

### 2.3. Ethical Approval

Ethical approval was obtained from the Health Research Ethics Committee of Stellenbosch University (Project ID 29142, Ethics Reference Number S24/03/054), and a waiver of consent was granted. This approval permitted a record review of all HIV‐infected patients who underwent a kidney biopsy at Tygerberg Hospital within the defined study period. All data were anonymised to ensure the privacy and confidentiality of participants’ personal information. The included histopathology images are anonymised and do not include any identifying patient information.

## 3. Results

### 3.1. Study Cohort

The cohort comprised 554 patients living with HIV who underwent native kidney biopsy between 1992 and 2020. Most biopsies (499/554, 90.1%) were performed after the rollout of ART (i.e., from 2004 to 2020). The mean age was 36.5 years, with a relatively equal distribution by sex. Mean systolic and diastolic blood pressures were 126 mmHg and 74 mmHg, respectively, and the median serum creatinine was 427 μmol/L. Patients in the post‐ART rollout era had lower levels of proteinuria (5.0 vs. 7.0 g/day). The median CD4 count was 193, and slightly more than half of patients were using ART. There were no statistically significant differences in baseline CD4 counts, serum creatinine concentrations, Hepatitis B serostatus, or proportions of patients using ARTs when stratified by period relative to ART rollout. The baseline clinicodemographic variables are described in more detail in Table 1.

**TABLE 1:** Baseline characteristics by period, relative to ART roll‐out.

	Pre–ART roll out (< 2004)	Post–ART roll out (≥ 2004)	Total	*p* value
*N*%	55 (9.9)	499 (90.1)	554 (100)	
Age (years), mean (SD)	34.9 (10.0)	36.7 (10.1)	36.5 (10.1)	0.238
SBP (mmHg), mean (SD)	122.5 (24.2)	126.8 (16.1)	126.0 (17.9)	0.150
DBP (mmHg), mean (SD)	79.6 (10.7)	72.8 (17.5)	74.1 (16.7)	0.012
Proteinuria (g/day)	7.0 [4.3–18.0]	5.0 [2.7–9.4]	5.1 [2.8–9.7]	0.005
Creatinine (μmol/L), median (IQR)	405.0 [141.0–1241.0]	428.0 [177.0–920.0]	427.0 [174.0–937.0]	0.569
CD4 (cells/μL), median (IQR)	294.0 [155.0–492.0]	191.0 [83.0–362.0]	193.0 [84.0–369.0]	0.169
Sex (missing/unknown excluded from analysis)				
Female, *n*%	20 (41.7)	257 (51.8)	277 (50.9)	0.179
Using ART? (missing/unknown excluded from analysis)				
Yes, *n*%	1 (100.0)	232 (54.8)	233 (55.0)	0.365
Hepatitis B serostatus (missing/unknown excluded from analysis)				
Yes, *n*%	3 (8.1)	32 (9.5)	35 (9.3)	0.787
Pathological group				
HIVAN only, *n*%	29 (52.7)	140 (28.1)	169 (30.5)	< 0.001
HIVAN with additional pathology, *n*%	4 (7.3)	80 (16.0)	84 (15.2)	
Non‐HIVAN pathology only, *n*%	22 (40.0)	279 (55.9)	301 (54.3)	

*Note:* ART, antiretroviral therapy; HIVAN, HIV‐associated nephropathy; IQR, interquartile range. *p* values < 0.05 were considered statistically significant.

Abbreviations: DBP, diastolic blood pressure; SBP, systolic blood pressure; SD, standard deviation.

**FIGURE 2:**
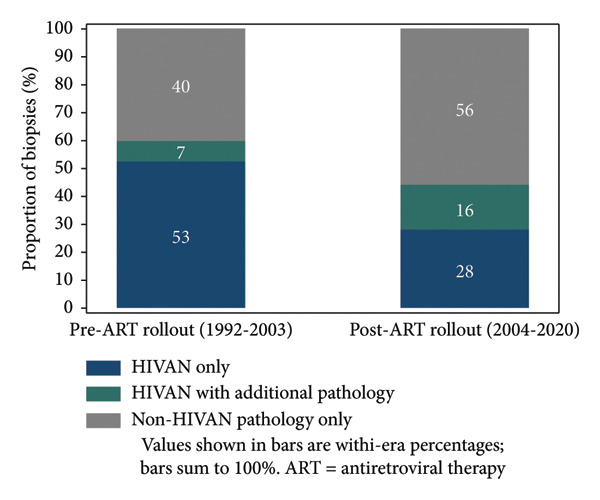
HIV‐associated kidney pathology by period.

**FIGURE 3:**
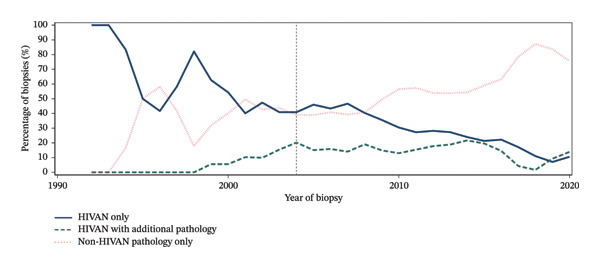
Trends in HIV‐associated kidney pathology, Tygerberg hospital (1992–2020). Lines represent three‐year moving averages; the vertical dashed line marks the antiretroviral therapy rollout (2004).

**FIGURE 4:**
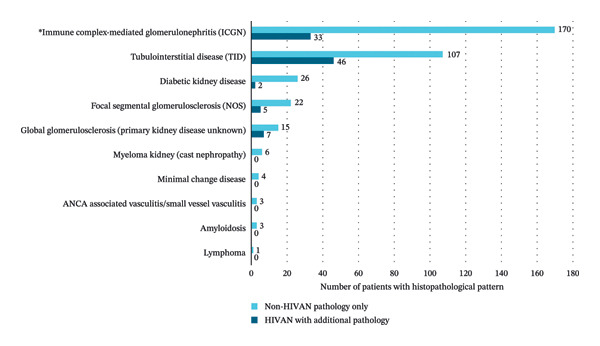
Spectrum of non‐HIVAN kidney pathology. ^∗^Immune complex glomerulonephritis includes mesangiocapillary glomerulonephritis, membranous nephropathy, postinfectious glomerulonephritis, IgA nephropathy, lupus nephritis, lupus‐like glomerulonephritis in the setting of HIV, mesangial proliferative glomerulonephritis with positive immunofluorescence, and any other immune complex glomerulonephritis. Tubulointerstitial disease includes tubulointerstitial nephritis and acute tubular necrosis from any cause, as well as pyelonephritis.

The overall prevalence of HIVAN in this cohort was (253/554, 45.7%), and one‐third of those with HIVAN (84/253, 33.2%) had an additional histopathological diagnosis. In the pre‐ART rollout era, HIVAN was the most prevalent diagnosis (33/55, 60.0%), whereas a lower prevalence (220/499, 44.1%) was observed post‐ART rollout. During the post‐ART rollout period, more than half of the patients (279/499, 55.9%) exhibited only non‐HIVAN pathology (see Figures 2 and 3). Among the 385 patients with any non‐HIVAN pathology (including those with non‐HIVAN pathology only and those with HIVAN with additional pathology), the most frequently observed non‐HIVAN histopathological pattern was immune complex‐mediated glomerulonephritis (ICGN) (203/385, 52.7%), followed by tubulointerstitial disease (TID) (153/385, 39.7%). The spectrum of non‐HIVAN lesions is summarised in (Figure 4).

### 3.2. Missing Data, Multiple Imputation and Sensitivity Analysis

Multiple variables in the pre‐ART rollout period had substantial missingness (> 50%); multiple imputation by chained equations (MICE) was therefore restricted to the post‐ART period. Tables S1–S3 (Supporting Information) detail the extent of missing data, variables included in the imputation model, and the imputed descriptive statistics for the post‐ART period.

Following multiple imputation, an exploratory multinomial regression limited to the post‐ART rollout period was conducted to examine the relationship between ART use and histopathological patterns, using non‐HIVAN pathology as the reference. ART use was associated with lower odds of isolated HIVAN (aOR = 0.39, 95% CI 0.24–0.64). In contrast, ART use was not associated with a difference in the odds of having HIVAN with additional pathology compared to non‐HIVAN pathology only (see Table S4, Supporting Information).

Delta‐method‐based patterns‐mixture sensitivity analysis was performed to assess the association between ART use and lower odds of having HIVAN, and findings remained consistent with the primary MICE and multinomial regression (Table S5, Supporting Information).

Complete case analysis significantly reduced the sample size, with only 87 patients (15.7%) having complete data, all from the post‐ART rollout era, preventing comparisons across eras.

## 4. Discussion

This study aimed to describe the patterns of kidney pathology in people living with HIV who underwent native kidney biopsy at Tygerberg Hospital between 1992 and 2020. The key findings were that HIVAN was the predominant kidney pathology (60.0%) in the pre‐ART rollout era, while most patients (55.9%) in the post‐ART rollout era had non‐HIVAN pathology only. Additionally, a greater proportion of HIVAN cases with additional pathology was observed in the period after ART rollout compared to the period before (16.0% vs. 7.3%). Although HIVAN was less common in the post‐ART rollout era, it still affected (44.1%) of patients during this time. The most observed non‐HIVAN histopathological patterns of kidney injury were ICGN and TID.

The finding of a greater proportion of patients with HIVAN in the pre‐ART rollout era compared to the post‐ART era is consistent with several studies conducted in South Africa. Diana et al. reported that, among kidney biopsies conducted in people living with HIV at two major teaching hospitals in Gauteng, the prevalence of HIVAN was 43.4% prior to ART rollout. In addition, they reported a lower prevalence (22.8%) in those biopsied during the post‐ART rollout period [13]. Similarly, in a study conducted at a tertiary hospital in Cape Town, Wearne et al. reported a decrease in the prevalence of HIVAN in the post‐ART rollout period (29%) compared with the period before ART rollout (45%) [14].

The proportion of patients with only non‐HIVAN pathology increased from 40.0% before ART rollout to 55.9% after ART rollout. Similarly, the proportion of patients with HIVAN coexisting with at least one other pathological diagnosis was higher (16.0%) in the post‐ART rollout period when compared to the period before rollout (7.3%). HIVAN is classically described in patients with severe immunocompromise (very low CD4 counts and unsuppressed viral loads) and, in the absence of ART, leads to the rapid development of kidney failure. Accordingly, those on ART are less likely to develop HIVAN. In addition, the use of ART in those with HIVAN leads to improvements in proteinuria and stabilisation of kidney function [3, 4, 6, 7, 9, 14]. In other words, patients on ART are more likely to have a longer lifespan and may develop kidney diseases other than or alongside HIVAN [6]. In our study, we demonstrated lower levels of proteinuria among patients in the post‐ART rollout period when compared to those in the pre‐ART period, possibly reflecting a lower prevalence of HIVAN. We did not observe a statistically significant difference in serum creatinine concentration between the two groups. In contrast, Diana et al. found that patients with HIV who were biopsied in the post‐ART era had better kidney function when compared to those biopsied before ART rollout [13]. Similarly, Wearne et al. reported lower serum creatinine concentrations in patients biopsied in the post‐ART rollout era [14]. In addition, they reported higher rates of virological suppression in the group biopsied post‐ART rollout [13, 14].

Although the proportion of patients with HIVAN decreased in the post‐ART rollout era, a significant proportion (44.1%) still had HIVAN. This is higher than the proportions of patients with HIVAN in the post‐ART period reported in the studies by Diana and Wearne et al. (22% and 29%, respectively [13, 14]). This likely reflects the fact that, even in the post‐ART rollout period, a significant proportion of patients in our study were not on ART. A report from the Western Cape Department of Health estimated ART usage among individuals living with HIV in the province at 59%, which was similar to the proportion of patients on ART in the post‐ART rollout period in our study (54%) [17]. Although we had significant missing data regarding ART use in the pre‐ART rollout group, a subgroup analysis of those biopsied in the post‐ART rollout era found an expectedly lower prevalence of HIVAN among those using ART. The reasons behind the relatively low proportion of patients living with HIV using ART despite its availability postrollout are complex and multifactorial. As per the Western Cape *U* = *U* Campaign Toolkit (2023), despite ongoing efforts to scale up HIV treatment services, the country remains behind in initiating people living with HIV on ART, leaving an estimated 2 million individuals untreated [17].

The most common non‐HIVAN histological pattern observed in this study was ICGN (52.7%), followed by TID (39.8%), with a higher proportion of patients with an additional diagnosis of TID in those with co‐existent HIVAN when compared to those with non‐HIVAN pathology only (55.4% vs. 35.5%). Immune complex‐mediated glomerulonephritis includes what was historically called HIV‐associated immune complex kidney disease (HIVICK). This term is regarded as vague in the literature, and there has been a move towards using more precise terminology. However, there is still considerable variation in what different sources include under this category and the patterns included under ICGN in this study broadly aligned with what KDIGO classifies as immune‐complex mediated glomerular disease [16]. The finding of ICGN as the most common non‐HIVAN pathological diagnosis in our cohort is unsurprising. Mesangiocapillary glomerulonephritis (MCGN), which was categorised under ICGN, was also the most frequently observed glomerular disease pattern in a study on biopsy‐proven kidney disease at Tygerberg Hospital [18, 19]. It is, however, important to acknowledge that MCGN only indicates a pattern of injury and is not exclusively immune‐complex mediated; it may also be complement‐mediated and can occur even with negative immunofluorescence [20]. Unless otherwise specified, MCGN in this study was assumed to be immune–complex‐mediated. In this study, TID comprised TIN and ATN from any cause. Both Wearne et al. and Diana et al. observed an increase in the prevalence of TID in the period post‐ART rollout [6, 13]. Although we lacked data on TDF use and ART duration, Wearne et al. identified an association between TID and TDF use, along with a significant number of patients with granulomatous interstitial nephritis caused by tuberculosis [6, 14]. Apart from TID, Diana et al. identified FSGS (NOS) as the most common non‐HIVAN diagnosis with HIV [13]. In our study, only 7.0% of patients had FSGS (NOS).

### 4.1. Strengths and Limitations

This study has several limitations. Because this is a retrospective, observational study, we were only able to determine associations and not causality. In addition, we encountered significant proportions of missing data for several variables. Since we obtained data from the institutional kidney biopsy registry, in many cases, we had access only to the final histopathological pattern, not the full biopsy report or the final clinical diagnosis. Unless otherwise specified in the summary report, the biopsies included were assumed to be adequate. There is likely some selection bias, since those biopsied are more likely to have had more significant manifestations of kidney disease, for example, elevated serum creatinine or significant proteinuria. Consequently, our findings may not be representative of all patients living with HIV and kidney disease. Additionally, survivorship bias might exist, as only individuals who lived long enough were biopsied—especially before ART rollout, when HIVAN was more common. The long study period may have reduced survivorship bias to some degree but may also have introduced temporal heterogeneity, as ART availability, clinical practice (e.g., biopsy thresholds), histopathological diagnostic techniques and classification systems have significantly evolved over the study period. Proteinuria was obtained from the registry without verification of the measurement method, which might have introduced nondifferential measurement error. Our study is strengthened by its inclusion of a large sample of people living with HIV who underwent kidney biopsy over nearly three decades at a tertiary academic centre with a high burden of HIV and considerable experience in evaluating kidney pathology in this population. Significant interobserver variation is reduced as all kidney biopsy findings are reviewed by a multidisciplinary team comprising a small, relatively consistent group of pathologists and nephrologists on a weekly basis, and this practice has been maintained throughout the study period. Two patients in the post‐ART period (2/499, 0.4%) underwent kidney biopsy in the first quarter of 2004. Given this very small proportion, this was considered unlikely to have meaningfully affected results. Furthermore, our approach of comparing broad pre‐ and post‐ART eras aligns with prior South African studies examining HIV‐related outcomes in relation to ART rollout [21]. We addressed missing data using multiple imputation with chained equations and sensitivity analysis and our key findings remained consistent.

## 5. Conclusion

This study described the spectrum of kidney pathology among patients with HIV at a tertiary centre in Cape Town, South Africa, over nearly three decades, demonstrating a substantial burden of HIVAN overall. Unsurprisingly, we observed a lower proportion of patients with HIVAN in the post‐ART rollout period compared to the period before ART rollout. An increase in the proportion of patients with non‐HIVAN pathology, most notably immune‐complex glomerulonephritis and tubulointerstitial disease, accompanied the decrease in the proportion of patients with HIVAN in the period after ART rollout.

## Author Contributions

Mogamat‐Yazied Chothia conceived the research idea. Mogamat‐Yazied Chothia and Thabiet Jardine provided the study design. Mogamat‐Yazied Chothia, Liezel Coetzee, Anna‐Liisa Tonata Kaapangelwa and Thabiet Jardine contributed to the data analysis. Thabiet Jardine performed the statistical analysis. Liezel Coetzee contributed to histopathological assessment and selection of included images (Figure 4). Anna‐Liisa Tonata Kaapangelwa drafted the initial manuscript. Each author contributed important intellectual content during the writing of the manuscript.

## Funding

No funding was received for this manuscript.

## Disclosure

All the authors approved the final version for publication and agreed to be accountable for the overall work.

## Ethics Statement

Ethical statement was granted by the Health Research Ethics Committee (HREC) of Stellenbosch University (HREC reference number: S24/03/054).

## Conflicts of Interest

The authors declare no conflicts of interest.

## Supporting Information

Additional supporting information can be found online in the Supporting Information section.

## Supporting information


**Supporting Information** Table S1. Missing variables summarised by time‐period. Table S2. Multiple imputation model. Table S3. Descriptive statistics for the post‐ART (2004–2020) time‐period—multiple imputation‐pooled; mean or proportions (%) with 95% confidence intervals (*N* = 499). Table S4. Multinomial regression. Table S5. Delta‐method based adjustment accounting for potential NMAR data.

## Data Availability

Data supporting this manuscript’s findings are available on request, subject to privacy and ethical restrictions.
