# Supplementary material for: The Spectrum of HIV‐Associated Kidney Pathology at a Tertiary Centre in South Africa
Source: Int J Nephrol. 2026 Jul 30;2026:3219794. doi: 10.1155/ijne/3219794 (PMC13424448; doi:10.1155/ijne/3219794)
Supplement: Supplementary file 1 — Supporting Information Table S1. Missing variables summarised by time‐period. Table S2. Multiple imputation model. Table S3. Descriptive statistics for the post‐ART (2004–2020) time‐period—multiple imputation‐pooled; mean or proportions (%) with 95% confidence intervals (N = 499). Table S4. Multinomial regression. Table S5. Delta‐method based adjustment accounting for potential NMAR data. [file IJNE-2026-3219794-s001.docx]

# **Supporting Information**

## **Table S1.** Missing variables summarised by time-period.

| **Variables** | **Pre-ART roll out (<2004),**  **n%** | **Post-ART roll out (≥2004), n%** | **Total, n%** |
| --- | --- | --- | --- |
| **Total** | 55 (9.9) | 499 (90.1) | 554 (100.0) |
| **Age** |  |  |  |
| Observed | 51 (92.7) | 494 (99.0) | 545 (98.4) |
| Missing | 4 (7.3) | 5 (1.0) | 9 (1.6) |
| **Sex** |  |  |  |
| Observed | 48 (87.3) | 496 (99.4) | 544 (98.2) |
| Missing | 7 (12.7) | 3 (0.6) | 10 (1.8) |
| **Systolic BP** |  |  |  |
| Observed | 46 (83.6) | 194 (38.9) | 240 (43.3) |
| Missing | 9 (16.4) | 305 (61.1) | 314 (56.7) |
| **Diastolic BP** |  |  |  |
| Observed | 46 (83.6) | 201 (40.3) | 247 (44.6) |
| Missing | 9 (16.4) | 298 (59.7) | 307 (55.4) |
| **Proteinuria (g/day)** |  |  |  |
| Observed | 25 (45.5) | 408 (81.8) | 433 (78.2) |
| Missing | 30 (54.5) | 91 (18.2) | 121 (21.8) |
| **Serum creatinine** |  |  |  |
| Observed | 55 (100.0) | 478 (95.8) | 533 (96.2) |
| Missing | 0 (0) | 21 (4.2) | 21 (3.8) |
| **CD4 count** |  |  |  |
| Observed | 7 (12.7) | 369 (73.9) | 376 (67.9) |
| Missing | 48 (87.3) | 130 (26.1) | 178 (32.1) |
| **Using ART?** |  |  |  |
| Observed | 1 (1.8) | 423 (84.8) | 424 (76.5) |
| Missing | 54 (98.2) | 76 (15.2) | 130 (23.5) |
| **Hepatitis B serostatus** |  |  |  |
| Observed | 37 (67.3) | 338 (67.7) | 375 (67.7) |
| Missing | 18 (32.7) | 161 (32.3) | 179 (32.3) |
| Rows show missing n (%) by variable and time-period.  Abbreviations**:** BP, blood pressure; ART, antiretroviral therapy. | | | |

**Table S2.** Multiple imputation model

| **Variable** | **Variable type** | **Imputation method** | **% missing ness** | **Predictor variables** |
| --- | --- | --- | --- | --- |
| **Age** | Continuous | Linear regression (regress) | 1.0% | All other imputed variables + diagnostic group; histopathological group (HIVAN only, HIVAN with additional pathology, non- HIVAN  pathology only); SBP and DBP as auxiliary variables only |
| **Sex** | Categorical | Logistic regression (logit) | 0.6% | Same predictors as above |
| **Serum creatinine** | Continuous | Linear regression (regress) | 4.2% | Log-transformed for purpose of regression,  back- transformed passively; same predictors as above |
| **Proteinuria (g/day)** | Continuous | Linear regression (regress) | 18.2% | Log-transformed for purpose of regression,  back- transformed passively; same predictors as  above |
| **CD4 count** | Continuous | Linear regression (regress) | 26.1% | Same predictors as above |
| **ART use** | Categorical | Logistic regression (logit) | 15.2% | Same predictors as above |
| **Hepatitis B serostatus** | Categorical | Logistic regression (logit) | 32.3% | Same predictors as above |
| Abbreviations**:** SBP, systolic blood pressure; DBP, diastolic blood pressure; ART, antiretroviral therapy. | | | | |

## **Table S3.** Descriptive statistics for the post-ART (2004-2020) time-period— multiple imputation-pooled; mean or proportions (%) with 95% confidence intervals (N=499)

| **Variable** | **Mean or Proportion (95% CI)** | **95% CI** |
| --- | --- | --- |
| **Age (years)** | 36.7 | 35.8–37.6 |
| **CD4 (cells/µL)** | 272.1 | 241.2–  303.1 |
| **Serum creatinine (µmol/L)** | 612.7 | 556.3–  669.1 |
| **Proteinuria (g/day)** | 8.2 | 6.8–9.5 |
| **Male sex (%)** | 48.2 | 43.7–52.6 |
| **Using ART (%)** | 54.5 | 49.7–59.2 |
| **Hepatitis B positive (%)** | 9.9 | 6.4–13.4 |
| Abbreviations**:** 95% CI, 95% confidence interval; ART, antiretroviral therapy. | | |

## **Table S4.** Multinomial regression

| **Predictor** | **HIVAN-only vs.non- HIVAN pathology only** | | **HIVAN with additional pathology vs. non-HIVAN pathology only** | |
| --- | --- | --- | --- | --- |
|  | **aOR [95%CI]** | **p-value** | **aOR [95%CI]** | **p-value** |
| **Age (years)** | 0.93 [0.92–0.96] | <0.001 | 0.9721 [0.9458–0.9992] | 0.04 |
| **Male sex** | 0.55 [0.34–0.89] | 0.02 | 1.08 [ 0.64–1.82] | 0.77 |
| **Serum creatinine (µmol/L, log-transformed)** | 1.0000 [0.9996–  1.0005] | 0.73 | 0.9999 [0.9993–1.0003] | 0.62 |
| **Proteinuria (g/day, log-transformed)** | 1.06 [1.02–1.10] | 0.01 | 1.04 [0.93–1.08] | 0.10 |
| **CD4 count (cells/µL)** | 0.9983 [0.9971–  0.9995] | 0.01 | 0.9995 [0.9983–1.0006] | 0.35 |
| **ART use** | 0.39 [0.24–0.64] | <0.001 | 0.61 [0.35–1.07] | 0.09 |
| **Hepatitis B** | 1.19 [0.50–2.84] | 0.70 | 0.57 [0.17–1.94] | 0.37 |

Abbreviations**:** aOR, adjusted odds ratio; 95% CI, 95% confidence interval; ART, antiretroviral therapy.

Near-1 aOR’s (0.97–1.03) rounded to four decimal places.

P-values <0.001 shown as “<0.001”); all other p-values rounded to two decimal places.

## **Table S5.** Delta-method based adjustment accounting for potential NMAR data.

| **Variable shift** | **Shift** | **aOR (95% CI) for ART use, HIVAN**  **only vs. non- HIVAN only** | **Interpretation (direction and magnitude)** |
| --- | --- | --- | --- |
| **MAR (base model)** | – | 0.39 [0.24-0.64] | – |
| **CD4 count** | +50 cells/µL | 0.42 [0.22-0.80] | Stable |
|  | -50 cells/µL | 0.42 [0.22-0.80] | Stable |
| **lnProteinuria** | +0.2 | 0.39 [0.24-0.63] | Stable |
|  | -0.2 | 0.40 [0.24-0.65] | Stable |

Abbreviations: NMAR, not missing at random; MAR, missing at random; aOR, adjusted odds ratio; 95% CI, 95% confidence interval; ART, antiretroviral therapy; HIVAN, HIV-associated nephropathy; lnProteinuria, log transformed proteinuria.

## **Complete case analysis**

Complete case analysis (CCA) led to a substantial reduction in the sample size, with only 87 patients (87/554, 15.7%) having fully observed data. Additionally, they were limited to the post-ART period, so no comparisons across different time periods could be made.
